# The LeFE algorithm: embracing the complexity of gene expression in the interpretation of microarray data

**DOI:** 10.1186/gb-2007-8-9-r187

**Published:** 2007-09-10

**Authors:** Gabriel S Eichler, Mark Reimers, David Kane, John N Weinstein

**Affiliations:** 1 Genomics and Bioinformatics Groups, Laboratory of Molecular Pharmacology, Center for Cancer Research, National Cancer Institute, National Institutes of Health, Bethesda, Maryland 20892, USA; 2 Bioinformatics Program, Boston University, Cummington St, Boston, Massachusetts 02215, USA; 3 Virginia Commonwealth University, Biostatistics Department, E Marshall St, Richmond, Virginia 23284, USA; 4 SRA International, Fair Lakes Court, Fairfax, Virginia 22033, USA

## Abstract

Interpretation of microarray data remains a challenge, and most methods fail to consider the complex, nonlinear regulation of gene expression. To address that limitation, we introduce Learner of Functional Enrichment (LeFE), a statistical/machine learning algorithm based on Random Forest, and demonstrate it on several diverse datasets: smoker/never smoker, breast cancer classification, and cancer drug sensitivity. We also compare it with previously published algorithms, including Gene Set Enrichment Analysis. LeFE regularly identifies statistically significant functional themes consistent with known biology.

## Abstract 2

The LeFE algorithm has been developed to address the complex, non-linear regulation of gene expression.

## Background

Data from microarrays and other high-throughput molecular profiling platforms are clearly revolutionizing biological and biomedical research. However, interpretation of the data remains a challenge to the field and a bottleneck that limits formulation and exploration of new hypotheses. In particular, it has been a challenge to link gene expression profiles to functional phenotypic signatures such as those of disease or response to therapy. A number of partial bioinformatic solutions have been proposed. The most mature and promising such algorithms have analyzed the data from the perspective of categories of related genes, such as those defined by the Gene Ontology (GO) or by the Kyoto Encyclopedia of Genes and Genomes [1]. Gene categories group genes into nonexclusive sets of biologically related genes by linking genes of common function, pathway, or physical location within the cell.

Gene categories introduce an independent representation of the underlying biology into the analysis of complex datasets and therefore serve to guide the algorithms toward conclusions congruent with conventional knowledge of biological systems. Algorithms that take such an approach have often demonstrated a higher level of functional interpretation than did earlier, single-gene statistical analyses. However, most gene category based methods still perform the analysis on a gene-by-gene, univariate basis, failing to capture complex nonlinear relationships that may exist among the category's genes. If, for example, upregulation of gene A influenced a drug sensitivity signature only if gene B in the category were downregulated and gene C upregulated, then that relationship would be missed. Here, we introduce a novel gene category based approach, the Learner of Functional Enrichment (LeFE) algorithm, to the interpretation of microarray (and similar) data. LeFE captures that type of complex, systems-oriented information for prediction of functional signatures.

The input to LeFE consists of the following components: signature vector, microarray (or analogous) data, and a predefined set of categories and the genes within them. The 'signature vector' describes the biological behavior, process, or state to be predicted for each experimental sample. The signature vector either classifies samples (for example, as normal or diseased) or assigns each sample a continuous value (for example, relative drug sensitivity). That is, the signature can be nominal or continuous. A discrete signature vector is handled as though it were continuous.

The goal of LeFE or any other gene category based algorithm is to determine which categories (for instance, molecular subsystems) are most strongly associated with the biological states described by the signature vector. Toward that end, most previously published methods, for example Gene Set Enrichment Analysis (GSEA) [2], assign each gene category a score based on nonparametric statistics, *t*-statistics, or correlations that reflect the relationships between individual genes and the signature vector. The gene categories most enriched with those strong single-gene associations are said to be related to the signature. The degree of enrichment is usually represented by a *P *value or false discovery rate using, for example, a Fisher's exact test [3,4], a weighted Kolmogorov Smirnov test [2], or comparison with a χ^2 ^[5], binomial [6], or hypergeometric [7] distribution. Although those approaches have proved useful, they neglect the fact that gene products generally function in complicated pathways or complexes whose expression patterns may not be reflected in the summation of univariate associations between single genes and the biological activity [8-11].

To address that shortcoming, LeFE uses a machine learning algorithm to model the genome's complex regulatory mechanisms, determining for each category whether its genes are more important as predictors (variables) than are a set of randomly sampled negative control genes. Although any of several different machine learning algorithms could be used in LeFE, we chose the Random Forest algorithm [12] because it has features (discussed below) that make it particularly apt for this application. The power of Random Forest has been successfully demonstrated in numerous bioinformatic and chemoinformatic applications [13-16]. As per the 'no free lunch' dictum [17], no single machine learning algorithm can be optimal for all datasets and applications, but Random Forest appears to be an appropriate choice as an engine for LeFE.

The Random Forest algorithm builds an ensemble of decision trees using the Classification and Regression Tree (CART) method [18]. Random Forest is therefore included among the general class of 'ensemble learning' algorithms. The algorithm injects diversity into the tree creation process by building each tree on an independently bootstrapped (resampled with replacement) subset of the samples. Further diversity among the trees is generated by basing each tree-split decision in each tree on a different randomly chosen subset of the variables. After the entire forest of slightly different decision trees has been built, it can be applied to new, unseen data by running each new sample down each tree. Just as in CART, each tree's ultimate classification or regression decision is determined by class voting on sample class or the median regression value of the training samples in the case of continuous variables. The aggregate forest's output is then determined by averaging the regression values of the trees or using a weighted voting process to determine the most common class decision reached by the trees. The power of random forests is derived from both the low-bias and the low-variability they achieve on the basis of the 'ensemble' of low-bias, high-variance decision trees.

At the simplest level, the Random Forest algorithm has only two tunable parameters: mTry, the fraction of all variables tried in each tree-split decision, and nTree, the number of trees grown. Typically in Random Forests, nTree is set to 500, but we used nTree = 400 since that choice showed no appreciable decline in the algorithm's accuracy and achieved a modest increase in efficiency. The best values of mTry, suggested by the literature [14], are *n*_*s*_/3 for regression on a signature vector with continuous values and √*n*_*s *_if the signature data contains class information, where *n*_*s *_is the number of experimental samples. We used those values, so there were no parameters that we tuned. The algorithm is therefore simple to deploy, and over-parameterization is relatively rare. The Random Forest algorithm also has two other properties that make it especially apt for use within LeFE. The first is that it includes an internal cross-validation procedure that estimates the forest's predictive performance without the need for explicit *a priori *separation of the testing and training samples. That feature is particularly important in this application because microarray experiments are often run on limited numbers of samples. Because each tree is constructed on a bootstrapped sample representing 1 - e^-1^, or approximately two-thirds of the samples, about one-third of the samples are not used to build any given tree. Those unused 'out-of-bag' (OOB) samples are unseen in training and therefore can be used to determine the predictive performance of the tree. After the forest is built, each sample serves as a test case for the approximately one-third of the trees for which it was OOB. That procedure provides an estimate of the forest's error in the prediction for each individual sample. The OOB error of each sample is averaged over all samples to estimate the total error of the model. Fivefold cross-validation and the internal performance assessment using OOB samples have been shown to yield quite similar results [14].

The second useful property of random forests is that they can determine the importance placed on each variable in the model. Each variable's importance is assessed by randomizing the variable's association (permuting the variable's row elements) with the samples and then reassessing the model's error by OOB cross-validation. The Random Forest software package, which we used for the computations, has one iteration as the default, and the documentation states that more than one randomization does not appreciably improve the stability of the calculated importance scores. The loss of model accuracy is normalized by the accuracy of the unpermuted, intact model's performance to give an 'importance score' for each gene in a category. When Random Forest is applied to a classification problem, the model's error is a weighted classification accuracy, and in the regression context model error is the mean squared error. The greater the decrease in normalized performance, the more instrumental was the variable (gene) in achieving the forest's predictive performance. See Materials and methods (below) for a detailed description of the importance score.

**Figure 1:**
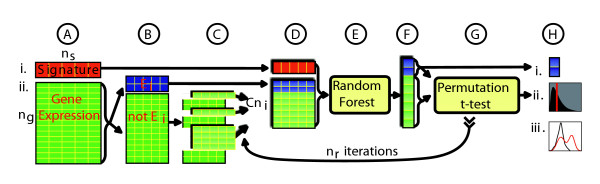
The LeFE algorithm illustrated schematically for a category of two genes. See Materials and methods for further details and Table 4 for a description of the steps (keyed to the circled letters). LeFE, Learner of Functional Enrichment.

The steps in the LeFE algorithm (shown schematically in Figure 1) are described more formally in the Materials and methods section (below). Here, we summarize the basic elements conceptually. For each category, LeFE builds a random forest to model the signature vector on the basis of a composite matrix consisting of genes in the category and a proportionately sized set of randomly selected negative control genes that are not in the category. On that basis, the random forest determines the importance score of each gene (variable) in the multivariate model. The distribution of importance scores of the genes in the category is then compared with the distribution of importance scores of the negative control genes. The expectation is that the two distributions will be similar when that comparison is made for a category that is biologically unrelated to the signature vector. However, if the category includes biologically relevant genes or gene combinations, then Random Forest is expected to assign higher importance scores to at least some of the genes. A one-sided permutation *t*-test [19] is used heuristically to compare the distribution of importance scores of the genes in the category with those of the negative control genes. Because the test compares the calculated *t*-scores with the distribution of such *t*-scores obtained after permuting the sample labels (instead of comparing them with a parametric *t*-distribution), it is nonparametric. To ensure diversity in the sampling of negative control genes, that process is repeated *n*_*r *_times, each with the same gene category and a different set of randomly selected negative control genes. As *n*_*r *_becomes large, the random gene sets asymptotically reflect the overall covariance of the dataset. The median of the permutation *t*-test's *P *values from the *n*_*r *_iterations is taken as an index of the degree of association between the gene category and the signature vector. After LeFE has been applied to each gene category, the categories are ranked according to those median *P *values.

LeFE is different from the other category-based algorithms listed previously [2,3,5-7] in that it assesses gene importance within the context of a multivariate model. That enables LeFE to access the gene information contained in complex biological interrelationships, rather than relying on the summation of univariate relationships within a category. For example, if two genes in a category were related to the samples' biological process or state by an 'exclusive OR' association, then LeFE could capture that relationship, whereas category-based summations of univariate associations would be likely to overlook it.

## Results

As proofs of principle we applied LeFE to three different prediction problems that represent diverse biological and computational scenarios. The first, current versus never-smoker classification, involvesIdentification of the molecular features that distinguish 57 current smokers from never-smokers on the basis of gene expression profiles of their lung epithelia [20]. The second problem, breast cancer classification, involves identification of characteristic molecular features that classify 49 primary breast cancer microarray samples as basal (estrogen receptor [ER] negative/androgen receptor [AR] negative), luminal (ER positive/AR positive), or 'molecular apocrine' (ER negative/AR positive) [21]. In the third problem, sensitivity to gefitinib, gene expression profiles are used to predict the gefitinib (Iressa, AstraZeneca, London, England) sensitivity of 26 non-small cell lung cancer cell lines. The continuous-valued signature vector consists of 26 log_10 _values of the 50% inhibitory concentrations [22].

### Gene categories

For use in all three applications, we assembled a set of 1,918 nonexclusive gene categories from multiple sources as follows. First, 1,396 gene categories were selected from the GO Consortium's biological process hierarchy. To ensure high quality of the categories, we removed those with evidence codes that denote lower quality assignments: inferred from electronic annotation, nontraceable author statement, no biological data available, and not recorded. Second, 522 gene categories, defined by the MSigDB v1 [2] collection of functional gene sets, was selected. Those categories had been assembled from various sources including BioCarta, GenMAPP, the Human Protein Reference Database, the Human Cancer Genome Anatomy Project, and a large number of manually curated publications.

For the analyses, we mapped the microarray gene annotations to categories and then included all categories in the broad size range from 2 to 150 genes. Because all of the studies used Affymetrix HG-U133A microarrays (Affymetrix Inc, Santa Clara, CA, USA), the mapping process was the same for all three datasets. That filtering process reduced the original set of 1,918 categories to a set of 1,282. Summaries of the 20 top-ranked categories for all three demonstration applications are given in Tables 1 to 3. Complete results for the three prediction problems, namely current versus never-smokers classification, breast cancer classification, and sensitivity to gefitinib, are available as Additional data files 1, 2, and 3, respectively.

**Table 1:** Top 20 LeFE Categories for current versus never-smokers classification

Rank	Category	FDR
1	Electron transporter activity BioCarta	~0
1	Carbohydrate metabolism (GO:0005975)	~0
1	Electron transport BioCarta	~0
1	Glutathione metabolism GenMAPP	~0
1	Pentose Phosphate Pathway BLACK	~0
6	Xenobiotic metabolism (GO:0006805)	0.016
6	O Glycans biosynthesis GenMAPP	0.016
8	PentosePathway BLACK	0.045
9	Protein amino acid O-linked glycosylation (GO:0006493)	0.069
10	Pentose phosphate pathway GenMAPP	0.094
10	Gamma hexachlorocyclohexane degradation GenMAPP	0.094
12	Tyrosine metabolism GenMAPP	0.097
13	Cysteine metabolism (GO:0006534)	0.105
13	G1pPathway BLACK	0.105
13	T cell differentiation (GO:0030217)	0.105
13	Fatty acid metabolism BioCarta	0.105
17	Retrograde vesicle-mediated transport, Golgi to ER (GO:0006890)	0.11
18	Aldehyde metabolism (GO:0006081)	0.11
19	Digestion (GO:0007586)	0.114
19	MAP00051 Fructose and mannose metabolism GenMAPP	0.114

FDR, false discovery rate; GO, Gene Ontology; LeFE, Learner of Functional Enrichment.

**Table 2:** Top 20 categories for breast cancer classification

Rank	Category	FDR
1	Breast_cancer_estrogen_signalling GEArray	0.02
1	Drug_resistance_and_metabolism BioCarta	0.02
1	FRASOR_ER_UP Frasor_et_al_2004	0.02
4	mta3Pathway BioCarta	0.041
5	Fatty_Acid_Synthesis BioCarta	0.065
6	Cell_cycle_checkpoint II	0.065
6	p35alzheimersPathway BioCarta	0.065
6	FRASOR_ER_DOWN Frasor_et_al_2004	0.065
9	L-phenylalanine catabolism	0.068
9	UDP-glucose metabolism	0.068
9	Cell_cycle_regulator	0.068
12	Electron_transporter_activity BioCarta	0.078
13	skp2e2fPathway BioCarta	0.1
14	Fatty_acid_metabolism BioCarta	0.1
15	Ubiquinone biosynthesis	0.102
16	MAP00010_Glycolysis_Gluconeogenesis GenMAPP	0.12
16	MAPKKK_cascade GO	0.12
18	G1Pathway BioCarta	0.134
18	MAP00280_Valine_leucine_and_isoleucine_degradation GenMAPP	0.134
20	Response to metal ion	0.144

FDR, false discovery rate; GO, Gene Ontology; LeFE, Learner of Functional Enrichment.

**Table 3:** Top 20 categories for sensitivity to gefitinib

Rank	Category	FDR
1	Androgen up genes na	0.347
2	EGF receptor signaling pathway BioCarta	0.408
3	MAP00100 Sterol biosynthesis GenMAPP	0.531
4	Epidermal growth factor receptor signaling pathway (GO:0007173)	0.531
5	G_1_/S transition of mitotic cell cycle (GO:0000082)	0.628
6	positive regulation of I-kappaB kinase/NF-kappaB cascade (GO:0043123)	0.628
7	Cell-cell adhesion (GO:0016337)	0.748
8	Aspartate catabolism (GO:0006533)	0.915
9	Calcium-independent cell-cell adhesion (GO:0016338)	0.915
10	Regulation of glycolysis (GO:0006110)	0.915
11	Detection of pest, pathogen or parasite (GO:0009596)	0.915
12	MalatePathway BLACK	0.915
12	RarPathway BLACK	0.915
14	Epidermis development (GO:0008544)	0.931
14	Regulation of endocytosis (GO:0030100)	0.931
16	NFKB reduced Hinata et al 2003	0.998
17	mRNA editing (GO:0006381)	~1
17	EMT DOWN Jechlinger et al 2003	~1
19	Chloride transport (GO:0006821)	~1
19	Induction of apoptosis by intracellular signals (GO:0008629)	~1

FDR, false discovery rate; GO, Gene Ontology; LeFE, Learner of Functional Enrichment.

### Current versus never-smoker classification

**Figure 2:**
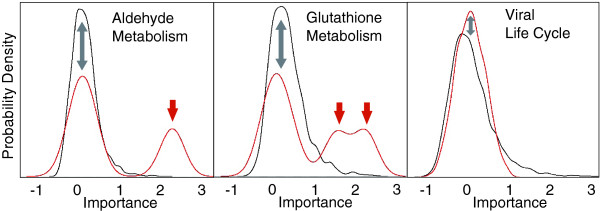
Importance plots (probability density distributions) of gene importance scores calculated by LeFE: smoker versus nonsmoker dataset. Shown are representative distributions for three gene categories (red curves) and their corresponding negative control gene sets (black curves). The curves were smoothed according to default settings of the 'density' function in R. The shifted secondary peaks, denoted by red arrows, for aldehyde metabolism and glutathione metabolism reflect genes important to the Random Forest models. The viral life cycle category contains no secondary peaks and therefore does not appear to be associated with smoking. See Results for further details.

Figure 2 shows what we term 'importance plots', which show the distribution of normalized importance scores of genes with respect to their prediction of the signature vector. The red and black curves represent the category's genes and the negative control genes, respectively. Each category is represented by a smoothed distribution, rather than a single value, because the curve represents importance scores calculated for all genes in all *n*_*r *_iterations of the Random Forest algorithm. The glutathione metabolism and aldehyde metabolism categories (positive examples) ranked among the top 20 categories, whereas the viral life cycle category (negative example) ranked 742th out of 1,282. Each of the two positive examples includes at least two peaks: one that corresponds to a peak in the negative control gene distribution (gray arrows) and one or more (red arrows) that reflect the biologically relevant genes. For example, the two top genes in aldehyde metabolism (aldo-keto reductase 1B10 and aldehyde dehydrogenase 3A1) have median importance scores in the peak denoted with a red arrow, and, as discussed below, they are known to metabolize cigarette smoke toxins [23]. The genes in the viral life cycle category are unrelated to smoking and have distributions indistinguishable from those of the negative control genes.

The highest-scoring five out of the 1,282 categories run through LeFE have median *P *< 0.001 (false discovery rate [FDR] < 0.02), and all of them contain genes that are known to exhibit altered expression in response to cigarette smoke *in vivo *or *in vitro*. Among the most important genes in the top-ranked category, electron transport, are *CYP1B1*, *CYP2A13*, and *MAOB*, all of which are known to be upregulated by cigarette smoke [24,25]. The top genes in the next category, electron transporter activity, include the aldo-keto reductases *AKR1B10*, *AKR1C1*, *AKR1C2*, and *AKR1C3*, as well as those encoding aldehyde dehydrogenase (*ALDH3A1*) and monoamine oxidase B (*MAOB*). In *vivo *studies have shown that those genes are upregulated in response to cigarette smoke condensate [23]. The third category, glutathione metabolism, fits with current understanding because glutathione, a tripeptide thiol antioxidant, forms conjugates with cigarette smoke toxins [26]. The fourth ranked category, pentose phosphate pathway, makes sense because, in response to blood plasma previously exposed to cigarette smoke *in vitro*, endothelial cells have been shown to release glutathione and activate the pentose shunt [27].

Among the top genes in the sixth ranked category, xenobiotic metabolism (FDR = 0.02), are *AKR1C1*, *CYP35A*, and *NQO1*. All three have independently been found to be differentially expressed in the bronchial epithelium of smokers [28,29]. Also in the same category is the gene that encodes UDP glucuronosyltransferase 1A6 (*UGT1A6*). Eight out of the 11 probes for that gene perfectly match the related *UGT1A7 *gene, which has been shown to detoxify multiple tobacco carcinogens [30]. Hence, the importance score for *UGT1A6 *may reflect a family resemblance in function, a cross-hybridization of probes, or both.

The 10th ranked category, γ-hexachlorocyclohexane degradation (FDR = 0.09), contains several cytochrome P450 genes with polymorphisms that are known to alter lung cancer risk for smokers. Furthermore, one of that category's highest scoring genes, *CYP1A1*, is expressed in primary lung cancer samples in a manner highly correlated with tobacco dose [31]. The 12th ranked category, tyrosine metabolism (FDR = 0.10), contains two previously mentioned aldehyde metabolism genes, *ALDH3A1 *and *MAOB*. The 16th ranked category, cysteine metabolism (FDR = 0.11), contains only two genes, namely *GCLC *and *GCLM*. Together they form the glutamate-cysteine ligase complex, which is responsible for increasing the antioxidant glutathione in the lungs of smokers [32].

We next compared LeFE directly with the popular and useful GSEA method [2]. The online documentation of that method suggests that GSEA should not be applied to categories smaller than 25 genes because such categories may produce inflated scores. Abiding by that limitation, GSEA would not have considered 13 of LeFE's top 20 categories, because they include fewer than 25 genes. However, for the sake of this comparison, we chose to ignore the 25-gene limitation and operate GSEA on all categories with a size of at least two. That resulted in a substantial overlap in the top 20 categories identified by LeFE and GSEA. However, several categories (including pentose phosphate pathway, aldehyde metabolism, γ-hexachlorocyclohexane degradation, and cysteine metabolism) that were ranked in the top 20 by LeFE were not in the top 140 categories as ranked by GSEA's FDR, despite the fact that they are all likely to be biologically related to cigarette smoke (see above and Figure 2). Furthermore, LeFE identified 44 categories with FDR below 0.2 and 150 categories with FDR below 0.5, whereas GSEA identified only 18 and 65, respectively. We cannot state definitively that LeFE did 'better' than GSEA at distinguishing the biology between the two sample classes, but the results do suggest that LeFE's unique method provides a different (although overlapping) set of categories that make considerable biological sense.

### Breast cancer classification

A dominant molecular characteristic of the breast cancer samples is ER-α (*ESR1*) status. Accordingly, the top categories identified by LeFE are intimately associated with that molecule and related subsystems. Three categories had median *P *values below 0.001 (FDR = ~0): breast cancer estrogen signaling; MSigDB's set of ER-upregulated genes identified by Frasor and coworkers [33]; and drug resistance and metabolism, which contains *ESR1*, *BCL2*, *AR *and ER's co-regulator *ERBB4 *[34]. The fourth category, the BioCarta-defined MTA3 pathway, contains *ESR1 *and three estrogen-regulated genes, namely *PDZK1*, *GREB1*, and *HSPB1 *(*HSP27*) [35] as the four most important genes.

Categories related to fatty acid synthesis and metabolism are represented three times in the top 25 categories, with FDRs below 0.02. That result is consistent with the observation that carcinomas of the colon, prostate, ovary, breast, and endometrium all express high levels of fatty acid synthase [36]. Manual literature searches failed to identify independently confirmatory research. However, we analyzed three independent breast cancer studies [37-39] on the Oncomine website [40] using conventional *t*-statistics and confirmed that many of the fatty acid related genes are, indeed, significantly differentially expressed among the three classes of breast cancers. Specifically, *PRKAB1*, *PRKAG1*, *PECI*, *CROT*, *FABP7*, and *ACADSB *levels were significantly higher in the ER-positive luminal class, whereas *PRKAA1 *levels were significantly higher in ER-negative samples. *FASN*, *FAAH*, and *SCN *were significantly lower in the AR-negative basal samples. The original publications on the datasets analyzed with LeFE noted the altered expression of metabolism genes but failed to identify that fatty acid metabolism systems are associated with breast cancer or breast cancer subtypes. The three categories related to fatty acid synthesis and metabolism contain various combinations of the aforementioned genes and interact with each other in complex ways that distinguish the breast cancer classes. GSEA does not handle multiclass analyses, at least directly, but even if it did it might well have overlooked the fatty acid categories because it depends on univariate associations between genes and sample class.

The three independent breast cancer datasets [37-39] from Oncomine also confirmed our findings for several other categories that had received top LeFE ranks and FDRs below 0.01. L-phenylalanine catabolism, cell cycle regulator, electron transporter activity, skp2e2f pathway, MAPKKK cascade, and response to metal ion (Table 2) contain many genes that received high LeFE importance scores in our LeFE analysis and were also significantly differentially expressed in those independent studies. Precise interpretation of the association between breast cancer and our independently verified genes, which include *GSTZ1*, *BCL2*, *MPHOSPH6*, *SRPK1*, *MCM5*, *BTG2*, *SKP2*, *DUSP7*, *NRTN*, *MTL5*, *NDRG1*, and *MT1X*, is beyond the scope of the present study. A direct comparison of results from GSEA [2] and LeFE for the breast cancer study was not possible because there were three classes.

### Gefitinib sensitivity

Gefitinib inhibits the tyrosine kinase activity of the epidermal growth factor receptor (EGFR) [41]. Accordingly, the second and fourth ranked out of 1,282 LeFE categories are the EGF receptor signaling pathway (FDR = 0.41) and EGFR signaling pathway (FDR = 0.53). If one is accustomed to a critical point such as 0.05 for *P *values, then an FDR of 0.53 may seem high. However, the implication is that almost half of the time such a category would constitute a true positive, rather than a false positive, even after correction for multiple hypothesis testing. Whether that level of certainty is high enough to act on depends, of course, on the relative cost and benefit of following up the finding. The predictions are clearly not as strong in the case of gefitinib as in the other two applications of LeFE presented here, but some of the top-ranked categories do make biological sense.

The first ranked category, androgen upregulated genes (FDR = 0.35), is interesting because there is evidence that androgen levels increase in non-small-cell lung cancer patients treated with gefitinib [42]. The third-ranked category, sterol biosynthesis (FDR = 0.53), assigns a high importance score to the gene that encodes 3-hydroxy-3-methyl-glutaryl coenzyme A (*HMGCR*). Gefitinib is synergistic with lovastatin [43], which inhibits *HMGCR *and is in clinical trials with simvastin, another *HMGCR *inhibitor, for treatment of non-small-cell lung cancer. That observation suggests the possibility of a link between the sterol biosynthesis pathway and gefitinib's activity.

The association between gefitinib and the fifth-ranked category, G_1_/S transition in mitotic cell cycle (FDR = 0.63), is not completely clear, but it has been shown that EGFR inactivity is required for G_1_/S transition in *Drosophila *[44]. The seventh category, cell-cell adhesion (FDR = 0.75), contains EGFR and Annexin A9, the latter being a cousin of the EGFR substrate Annexin A1. That could represent a novel finding or be due to cross-hybridization of the microarray's probes. A comparison of LeFE and GSEA was not possible because GSEA [2] does not operate directly on continuous valued signature vectors.

## Discussion

LeFE is a novel statistical/machine learning method for functional analysis of microarray (and analogous) data. Here, we have implemented it using the Random Forest algorithm with internal cross-validation. LeFE's attention to gene categories differentiates it from earlier microarray analysis methods based on individual genes (for instance, correlation analysis or *t*-tests). Its ability to model complex relationships among the genes within a category also differentiates it from previous category-based ((hyphen necessary to meaning))algorithms (for example, GSEA and methods based on the Fisher's exact test) that are founded on summation of the univariate effects of individual genes within a category. Needless to say, the ability to build more complex models carries with it a potential cost, namely that of 'over-fitting'. However, LeFE's use of negative control gene sets and internal cross-validation mitigate that concern considerably, and the three proof-of-principle applications described in the Results section speak for themselves. We would not claim that LeFE is 'better' than previous useful methods such as GSEA, but it does clearly have independent value, and it does directly handle problem types (multi-class, continuous valued signature, small categories) that are not handled directly by the other methods.

Our application of LeFE to gene expression in the lung epithelia of current smokers, as opposed to never-smokers, demonstrated its ability to identify and elucidate molecular differences between two sample classes. LeFE correctly identified categories containing the glutathione related genes, aldehyde dehydrogenases, monoamine oxidase, several aldo-keto reductases, and cytochrome P450 genes, all of which are differentially expressed in response to cigarette smoke or in the lungs of smokers. Four of the top biologically important categories were overlooked by GSEA, thereby highlighting LeFE's independent value.

However, a cautionary consideration is in order. Given the vast searchable archives of published biological research, it seemed possible that identifying literature citations consistent with LeFE's findings had a high *a priori *probability or that it was tainted by multiple hypothesis-testing. To address those possibilities, we designed a simple blinded experiment to test how well LeFE performed in the eyes of a pulmonology expert, Dr Avrum Spira, lead researcher on the lung epithelium gene expression study and first author of the resulting article [20]. We presented him with the top 20 gene categories identified by LeFE, each of them matched with a randomly chosen category of identical, or essentially identical, size. Because some categories have vague names, we also provided the names of the five most important genes in each category. We then asked Dr Spira, who was blinded to the LeFE results, to identify which category in each pair was more likely to be associated with gene expression differences in the epithelium of smokers as opposed to nonsmokers. He correctly distinguished the top seven categories and 17 of the top 20 from their size matched, randomly chosen counterparts. The binary probability of achieving at least 17 out of 20 correct by chance is *P *< 0.0002. An additional, independent application of LeFE to the same dataset yielded an overlap of 17 out of the top 20 categories. All three of the new results were correctly identified by Dr Spira.

Our additional applications of LeFE, to gene expression in three breast cancer classes and to *in vitro *gefitinib sensitivity (see Results), provide further proofs of principle. The findings highlight the distinctions between LeFE and the univariate category based methods. They also underscore the utility of LeFE's novel 'importance plots' for relating the individual gene importance scores to complex relationships within a category.

**Figure 3:**
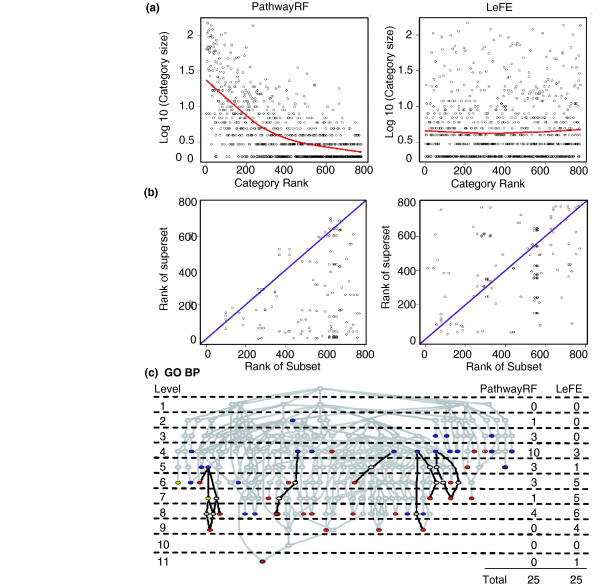
A Comparison of LeFE with PathwayRF Shown is a comparison of Learner of Functional Enrichment (LeFE) and PathwayRF with respect to the size distribution of categories identified as important for breast cancer classification using the Gene Ontology (GO) biological process categories. **(a) **Scatter plots showing category rank versus category size. Ties in category ranks were resolved through random reordering. Red lines are lowess regressions. **(b) **Comparison of GO superset and subset ranks. Almost all points for PathwayRF are below the blue x = y line, indicating that supersets rank lower (better) than that their corresponding subsets. The panel for LeFE shows no such bias. **(c) **The GO biological process hierarchy (with the most general categories toward the top). Blue circles denote the top 25 categories ranked by PathwayRF; red circles denote the same for LeFE; and yellow circles denote categories in the top 25 for both algorithms. The mean GO level is 4.92 for PathwayRF and 7.08 for LeFE. There are no cases in which LeFE's top results are the ancestors of top results from PathwayRF. However, the black edges highlight eight cases in which LeFE found categories that are progeny of categories identified by PathwayRF.

LeFE's hybrid machine learning/statistical algorithm compares gene categories with sets of randomly selected negative control genes. That approach distinguishes LeFE from the superficially similar PathwayRF [45] program, which was recently reported during the preparation of this paper. The PathwayRF algorithm trains a single random forest on each gene category's genes and then ranks the categories according to the model's predictive accuracy. Unlike LeFE, PathwayRF does not use gene importance scores at all. Results presented in the PathwayRF report indicate that it can provide biologically meaningful insight into gene microarray datasets, but the algorithm has a hidden bias that favors large categories. The predictive power of a statistical or machine learning model increases as independent variables are added if no penalty is imposed for adding those variables, and PathwayRF does not impose such a penalty. Therefore, as shown in Figure 3a, it strongly favors large gene categories because they contain more variables (genes). The mean and median numbers of genes in the top 20 categories for PathwayRF are 68 and 36, respectively. The corresponding values for LeFE are 32 and 22.

PathwayRF's bias toward larger categories can be demonstrated most concretely, as shown in Figure 3b, by considering the frequently occurring superset-subset (nested) relationships between gene categories in the hierarchically organized GO. With PathwayRF, the superset of a nested category's model is essentially guaranteed to exhibit predictive power at least as great as that of any nested subcategory; all models that can be generated by the subset can also be generated by the superset. (The few points above the diagonal line for PathwayRF in Figure 3b are probably there by chance because the algorithm is stochastic in nature.) However, as shown in Figure 3c, even when there is no nesting, larger and more biologically diffuse categories are much more likely to do better than smaller, more specific ones. Methods that favor a general hypothesis over a more specific one are likely to mis-prioritize follow-up studies. Therefore, any method in the spirit of LeFE or PathwayRF must correct for category size, and LeFE does that by using a set of negative control genes proportional in size to that of the category.

## Conclusion

In conclusion, we have presented LeFE, a novel statistical/machine learning algorithm for interpretation of microarray (and analogous) data. LeFE exploits information related to the complex, interactive regulation of gene expression and does not suffer from bias toward large category size. We have demonstrated LeFE's value on three diverse datasets and have shown that the results are either consistent with independently determined biological conclusions or generate novel, plausible hypotheses. A comparison of results from LeFE and GSEA suggests that LeFE identifies important biological information overlooked by the latter method, which does not take into account the complex interrelationships among genes within a category. A new type of visualization, the 'importance plot', captures the distribution of importance scores within a category. Unlike GSEA [2], LeFE is directly applicable to problems with multiple classes or continuously valued signature vectors. A user-friendly program package, LeFEminer, is freely accessible on the internet [46].

## Materials and methods

### Technical description of LeFE

#### Input

Figure 1 shows a schematic flow diagram of the LeFE algorithm. The first input(indicated by i in Figure 1) is a vector *Y *of *n*_*s *_sample signature values, each representing a behavior, phenotype, or state of the sample. The signature values may denote classes of samples (for example, for the three breast cancer categories) or continuously distributed values (for example, drug sensitivity). The second input (denoted ii in Figure 1) is a matrix *X *of gene expression values for *n*_*g *_genes measured over the *n*_*s *_samples. The third input (not shown in Figure 1) is a set *E *of *m *gene categories {*E*_1_, *E*_2 _... *E*_*i *_... *E*_*m*_}. Each category *E*_*i *_contains *n*_*i *_genes predetermined to be functionally related. Categories can, for example, be GO categories [47] or Kyoto Encyclopedia of Genes and Genomes pathways [1].

#### LeFE algorithm

The LeFE algorithm assigns a score that indicates the category's predicted biological association with *Y*. The steps in the algorithm, as applied to a single category, are listed in Table 4, which is keyed to the circled letters in Figure 1.

**Table 4:** Steps in the LeFE algorithm

Step	Details
A	The gene expression matrix, signature vector, and gene categories (not shown in Figure 1) are entered
B	For each category *E*_*i*_, the genes in the microarray (*X*) are split into those in *E*_*i *_and those not in *E*_*i *_(denoted, respectively, by two blue and 12 green rows of the gene expression matrix)
C	A negative control set consisting of *C *× *n*_*i *_genes in *X *but not in *E*_*i *_is selected at random. Those genes are noted as elevated rows in Figure 1, which was arbitrarily drawn for *C = *3. The integer constant *C *is used to mitigate issues of statistical imprecision associated with small categories. The default value *C = *6 creates better run-to-run reproducibility and was used in the actual LeFE calculations
D	A composite matrix of gene expression *X*_*iter *_is assembled by selecting the rows in *X *that correspond to the *n*_*i *_genes in *E*_*i *_along with the negative control genes selected in step C. The resulting data structure, *X*_*iter*_, has dimension (C × *n*_*i*_) + *n*_*i *_rows by *n*_*s *_columns
E	A random forest with 400 trees is built on *X*_*iter *_to model the signature vector, using the default value for the random forest parameter mTry. The random forest is given no information for distinguishing between category genes and negative control genes
F	A vector *I *of standardized importance scores of each gene in *X*_*iter *_is computed internally from the random forest. *I *is then divided into two sets of importance scores, *I*_*E *_and *I*_*notE*_, for the genes in *E*_*i *_and the negative control set, respectively
G	The statistical significance of the gene expression evidence for rejection of the null hypothesis that the mean of *I*_*E *_is less than or equal to the mean of *I*_*notE *_(that the category genes are, on average, more important to the Random Forest model than the negative control genes) is determined by a one-sided permutation *t*-test. Because that test compares observed *t*-statistics with a null distribution of *t*-statistics of permuted data, it avoids using the parameterized *t*-distribution and is therefore nonparametric. For statistical robustness, steps C to G are repeated *n*_*r *_times with different, randomly selected sets of negative control genes. The covariate structures of the *E *and *notE *genes are likely to differ, but the negative control genes, selected at random from *notE*, are unbiased with respect to the ranking of categories in the next step and with respect to the calculation of false discovery rates
H	Applying the above procedure to a single gene category creates three outputs. The first (denoted *i*, in Figure 1) is a median importance score for each of the *n*_*i *_genes in the category. Because the median importance scores are computed within the category's multivariate Random Forest models, they reflect the genes' importance within its complex biological context. The second output (denoted *ii*) is the entire category's median *P *value from the *n*_*r *_permutation *t*-tests. The third output (denoted *iii*) is an importance plot, which compares the distributions of importance scores of the genes in the category and the negative control genes

Shown are the steps in the Learner of Functional Enrichment (LeFE) algorithm, with the letters in the left-hand column corresponding to those in Figure 1.

#### Output

The results (not shown in Figure 1) of applying the algorithm to all categories are as follows: a sorted vector of length *m*, representing the ranked median permutation *P *values of the *m *gene categories; an importance score for each gene in the context of each category in which it occurs; and an importance plot (provided only for top categories), which shows the distribution of importance scores for all genes in all *n*_*r *_iterations (Figure 2).

### Estimation of statistical significance

The FDR associated with each gene category's median permutation *t*-test value is estimated by permuting the signature vector and calculating the fraction of more extreme scores for data that contain no true biological information. For each of the example analyses described in this report, we have computed FDRs using the method described by Benjamini and Hochberg using 50 independent signature vector permutations [48].

### Importance scores

Gene importance scores were described in general terms in the Introduction (above). A more formal description, adapted from Breiman and Cutler [49], is provided here. For each (microarray) sample *i *in our experiment, let X_*i *_represent the vector composed of gene expression values of the category's genes and its randomly selected negative control gene set. Let *y*_*i *_represent the sample's true classification or regression value, let *V*_*j*_(X_*i*_) be the vote of tree *j *when trained on the values contained in X_*i*_, and let *t*_*ij *_be an indicator variable equal to 1 if *i *is an OOB sample for tree *j *and otherwise 0. Let X^(*A*,*j*) ^= (X_1_^(*A, j*)^, ..., X_*N*_^(*A, j*)^) represent the gene expression values with the value of gene *A *randomly permuted among the OOB observations for tree *j*. Then, X^(*A*) ^is the collection of X^(*A*,*j*) ^for all trees, where *N *samples have been selected with replacement from the study's set of *n*_*s *_experimental samples. This notation can easily be used to define importance scores in both the classification and regression contexts if we define the function *f*(α,β). In the context of classification, *f = 1 *if α is logically equal to β and is otherwise 0. In the context of regression, *f *is the mean squared difference between α and β. Thus, the importance score, *I*_*T*_, of variable *A *is defined as follows:

$$I_{T}(A)=\frac{1}{T}\sum_{j=1}^{T}\frac{1}{N_{j}}\sum_{i=1}^{N}\left[f\left(V_{j}\left(X_{i}\right),y_{i}\right)-f\left(V_{j}\left(X_{i}^{\left(A,j\right)}\right),y_{i}\right)\right]t_{ij}$$

where *T *is the total number of trees in the forest and *N*_*j *_represent the number of OOB samples for the *j*th tree. It is then straightforward to see that if the variable *A *is unimportant and therefore infrequently used, *f *(*V*_*j *_*X*_*i*_, *y*_*i*_) ≈ f(*V*_*j *_$X_{i}^{\left(A,j\right)}$) and *I*_*T*_(*A*) ≈ 0.

Importance plots show the distribution of importance scores normalized by the standard error of the inter-tree variances of *I*_*T*_(A), $\sigma_{T}^{2}$. Therefore, the normalized importance scores are as follows:

ZT(A)=IT(A)σT2(A)T

The importance plot shows two smoothed probability density distributions of importance scores: one for the genes in the category (red curve) and the other for the sets of negative control genes (black curve). If category *i *has |*E*_*i*_| genes, then the red distribution (of the category's genes) is composed of *n*_*r*_*× *|*E*_*i*_| genes, and the black distribution (with corresponding negative control genes) has *n*_*r*_*× *|*E*_*i*_| × *C *genes, where *C *is an integer constant defined in Table 4 (step C).

Importance scores are usually positive values, but it is possible for them to take on negative values if the model's performance happens to improve when the variable is permuted.

Because importance scores are computed within the model's context, they reflect the complex, multivariate relationships among genes in a category, even though they are assigned to individual genes. That duality is key to the LeFE algorithm.

### Implementation of LeFE

We implemented the algorithm in R v2.4 [50], using Bioconductor [51], and the randomForest R package. Multiprocessor capabilities in the Rmpi and snow R libraries were used to speed up LeFE's extensive computations. A user-friendly, Java-based web application, LeFEminer, is freely available on the internet [46]. It enables nontechnical experimentalists and biologists to apply the algorithm to their datasets. To perform LeFE's heavy computational tasks, the web service application is multiprocessed on six 1.5 GHz 64-bit Itanium processors with 4 GB RAM at the National Cancer Institute's Advanced Biomedical Computing Center. Analysis of a typical microarray experiment takes 90 to 240 min.

**Figure 4:**
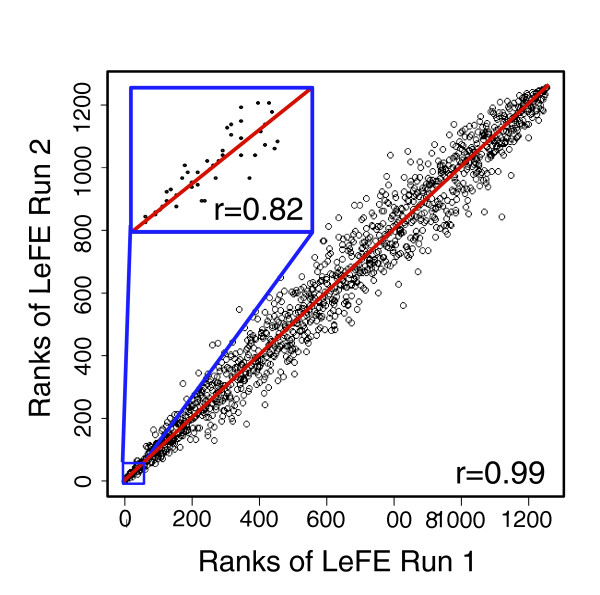
Replicate applications of LeFE to the breast cancer classification dataset. Scatter plot comparing the ranks resulting from two applications of Learner of Functional Enrichment (LeFE) to the breast cancer classification dataset, with *n*_*r *_= 75, *n*_*c *_= 6, and *nTree *= 400. The inset represents a blowup of the top 50 categories. *r *denotes the Pearson's correlation coefficient of the ranks (the Spearman correlation coefficient).

To manage the high computational requirements of running the LeFE algorithm, and because most users are most interested in the top-ranked gene categories, a slightly modified version of LeFE is supported by the LeFEminer web application [46]. Median permutation *t*-test *P *values are rapidly estimated by the LeFE algorithm with *n*_*r *_= 10 iterations instead of the more accurate (and time consuming) *n*_*r *_= 75 iterations. Based on that preliminary ranking of each category's likelihood of being important, LeFEminer prioritizes the calculation, running up to *n*_*r *_*= *75 iterations for the top categories and fewer for the less promising ones according to a logistic curve. Using the logistic curve, LeFE sets *n*_*r *_to be at least 60 for the top 40 preliminarily ranked categories and then gradually reduces *n*_*r *_until it settles at 20 for the least promising (roughly 85%) of the categories. That slight modification preserves the accuracy and reproducibility of the results for the top ranked categories while using only a fraction of the computation time. LeFEminer uses the increased efficiency to estimate the FDR of the results. Replicate applications of the faster version of LeFE to the three datasets analyzed (current versus never-smokers, breast cancer type, and gefitinib sensitivity) produced highly reproducible rankings, with Spearman correlation coefficients (calculated over all of the ranks) ranging from 0.95 to 0.98 (see Figure 4 and Table 5).

**Table 5:** Correlation of ranks between two applications of LeFE with different random number generator seeds

Dataset	Overall correlation	Top 50 correlations
Breast cancer	0.98	0.70
Current Smoker/Never- Smoker	0.97	0.70
Gefitinib	0.95	0.61

LeFE, Learner of Functional Enrichment.

### Reproducibility of LeFE

Because LeFE is not a deterministic algorithm, reaching full computational convergence may require relatively high values of *n*_*r*_and *n*_*c*_, along with random forests of considerable size. We used *n*_*r *_= 75, *n*_*c *_= 6, and *nTree *= 400, respectively, in the present calculations. Figure 4 shows the reproducibility of LeFE for the breast cancer classification dataset. The overall Spearman correlation over replications was *r *= 0.99, and the correlation of the top 50 categories was *r *= 0.82. The results of repeated analyses using the more efficient LeFEminer implementation (see above) on all three datasets are summarized in Table 5.

### Data for use in LeFE

LeFE and its internal Random Forest algorithm can be operated on a variety of prediction problem types. Unlike GSEA [2], it directly handles regression and multi-class classification. However, its flexibility creates a limitation worth discussion. LeFE's Random Forest algorithm requires a collection of samples that exemplify diverse states or behaviors. The default setting of the randomForest R package used by LeFE grows each decision tree until the tree's leaves have no fewer than five samples in a regression model or one sample in a classification model. Because the goal of LeFE is to capture any gene interactions, the simplest logical models not captured by standard univariate methods must contain at least two genes. Despite the observation that random forests consider only a subsampling of variables to make each decision tree split, LeFE can, in fact, be applied to small categories with as few as two variables (genes). That is so because the random forests are actually constructed on composite categories of at least 2*n*_*c *_+ 2 = 14 genes in the case of a category with two genes and *n*_*c *_= 6. As an informal test of whether LeFE was doing something reasonable with small gene categories, we examined the highest ranked two-gene categories (T cell differentiation and cysteine metabolism) in the current-smoker/never-smoker dataset. In each case, the low permutation *t*-test *P *values were explained by the observation that both genes in the category had high importance scores.

## Abbreviations

AR, androgen receptor; CART, Classification and Regression Trees; EGFR, epidermal growth factor receptor; ER, estrogen receptor; FDR, false discovery rate; GO, Gene Ontology; GSEA, Gene Set Enrichment Analysis; LeFE, Learner of Functional Enrichment; OOB, out-of-bag; RF, Random Forest.

## Authors' contributions

GE formulated the original concept of LeFE and implemented the R scripts; he also ran all of the demonstration calculations, built the website's entire back-end and parts of the front-end, and wrote the most of the manuscript. MR consulted on statistical aspects of the LeFE algorithm. DK built the web application's front-end and middle-tier communications layers. JNW consulted on the algorithm and biological results and also helped to write the manuscript.

## Additional data files

The following additional data are available with the online version of this paper. Additional data file 1 provides results from the current/never-smoker demonstration. Additional data file 2 provides complete results from breast cancer demonstration. Additional data file 3 provides complete results from the gefitinib demonstration.

## Supplementary Material

Additional data file 1 Complete results from the smoker/never-smoker demonstration, including gene categories ranked by LeFE computed median permutation *t*-test *P *value and individual gene importance scores. Click here for file

Additional data file 2 Complete results from the breast cancer demonstration, including gene categories ranked by LeFE computed median permutation *t*-test *P *value and individual gene importance scores. Click here for file

Additional data file 3 Complete results from the gefitinib demonstration, including gene categories ranked by LeFE computed median permutation *t*-test *P *value and individual gene importance scores. Click here for file
